# Supplementary material for: Endogenous nicotinamide riboside metabolism protects against diet-induced liver damage
Source: Nat Commun. 2019 Sep 20;10:4291. doi: 10.1038/s41467-019-12262-x (PMC6754455; doi:10.1038/s41467-019-12262-x)
Supplement: Supplementary file 1 — Supplementary Information [file 41467_2019_12262_MOESM1_ESM.pdf]

SUPPLEMENTARY INFORMATION

Endogenous Nicotinamide Riboside metabolism protects against diet-induced liver damage

Sambeat A., Ratajczak J., et al.

CONTENTS:

Supplementary Figure 1 (Page 3)

Supplementary Figure 2 (Page 4)

Supplementary Figure 3 (Page 6)

Supplementary Figure 4 (Page 8)

Supplementary Figure 5 (Page 10)

Supplementary Figure 6 (Page 12)

Supplementary Figure 7 (Page 14)

Supplementary Figure 8 (Page 15)

Supplementary Figure 9 (Page 16)

Supplementary Figure 10 (Page 17)

Supplementary Table 1 (Page 18)

Supplementary Table 2 (Page 19)

Supplementary Methods (Page 20)

Supplementary References (Page 26)

Supplementary Figure 1

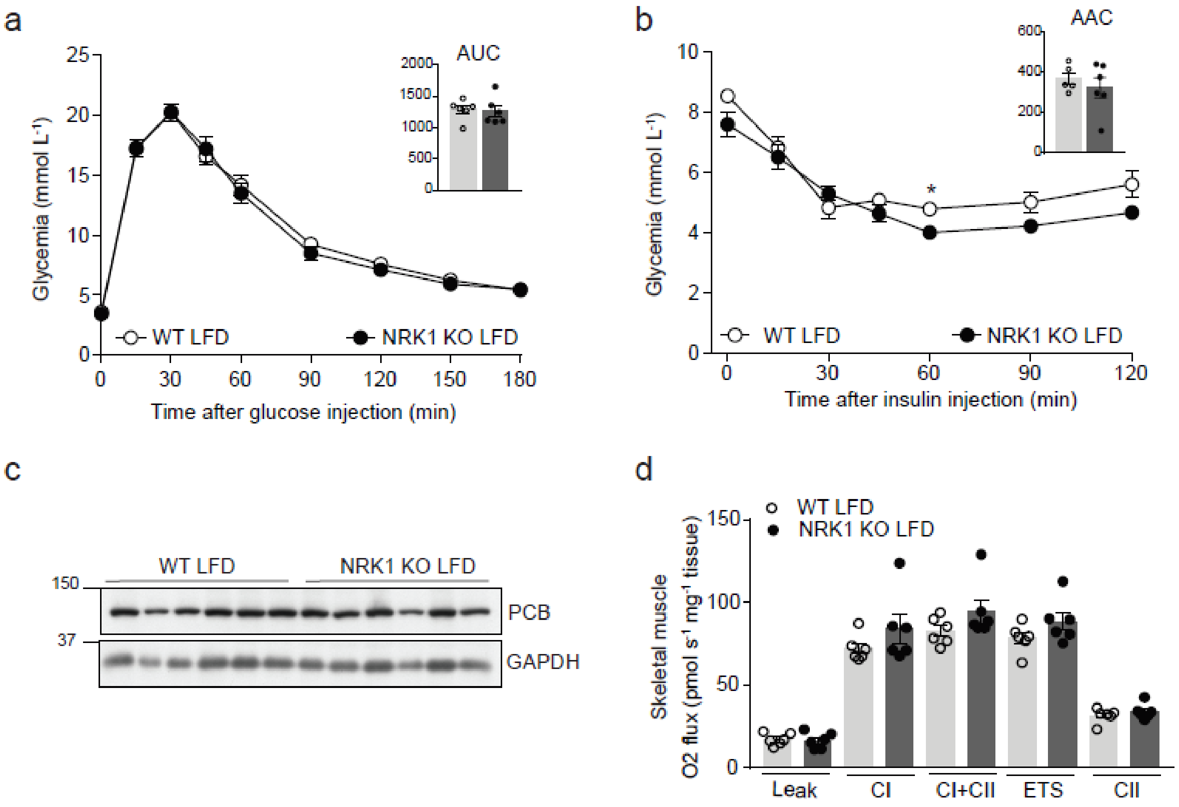

Supplementary Figure 1: NRK1 KO mice display lower gluconeogenic capacity

NRK1 KO and controls (WT) mice were fed with a low-fat diet (LFD). **a**. Blood glucose level during intraperitoneal glucose tolerance test (n=6 mice per group). **b**. Blood glucose level during intraperitoneal insulin tolerance test (n=5 for WT mice, n=6 for KO mice). **c**. Protein level of Pyruvate carboxylase (PCB) and GAPDH in the liver of NRK1 KO and control (WT) mice (n=6 mice per group). **d.** Mitochondrial respiration in the soleus muscle of NRK1 KO and control mice. (n=6 mice per group). Results shown are mean ± SEM; * $p<0.05$ ** $p<0.01$ *** $p<0.001$. The individual values and statistical tests used for each panel can be found in the Data Source file.

Supplementary Figure 2

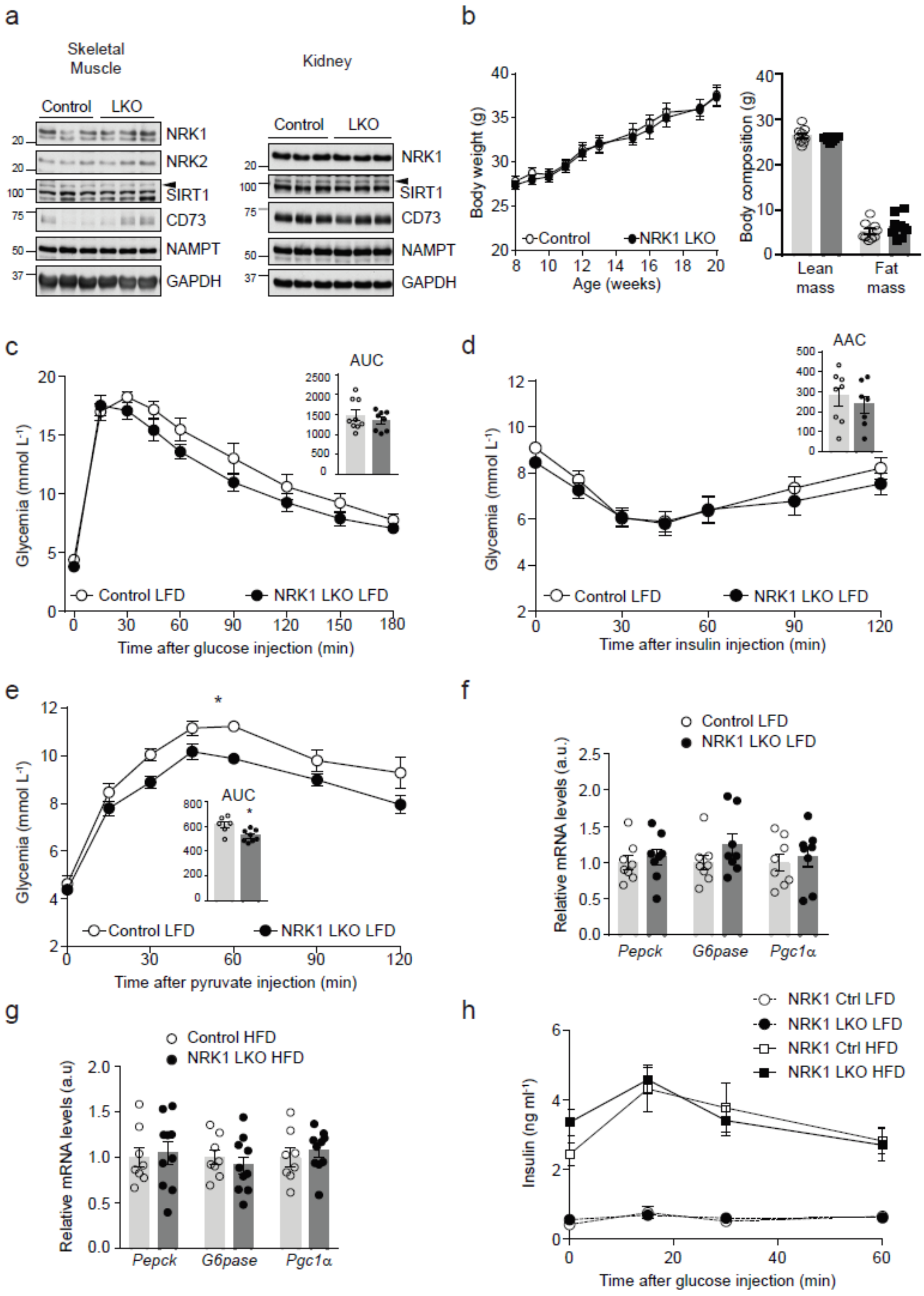

Supplementary Figure 2: Liver-specific NRK1 deficiency impairs gluconeogenesis .

a. Protein level of NRK1, NRK2, SIRT1 (arrow), CD73, NAMPT and GAPDH in skeletal muscle (left) and kidney (right) of NRK1 LKO and control mice (n=3 mice per group). **b.** Body weight and body composition of NRK1 LKO (n=8) and control (n=9) mice on LFD. **c-d**. Blood

glucose level during intraperitoneal glucose tolerance test (**c**) and intraperitoneal insulin tolerance test (**d**) for NRK1 LKO (n=8) and control (n=9) mice on LFD. **e**. Blood glucose level during intraperitoneal pyruvate tolerance test in NRK1 LKO (n=8) and controls (WT; n=6) mice on LFD. **f-g**. Expression of gluconeogenic genes in NRK1 LKO and control littermates on LFD **(f)** and HFD (n=8 mice per group) **(g)**. **h**. Plasma insulin level during intraperitoneal glucose tolerance test for NRK1 LKO and control mice upon LFD (n=7 mice per group) and HFD (n=8 mice per group). Results shown are mean ± SEM, * $p<0.05$ ** $p<0.01$ *** $p<0.001$. The individual values and statistical tests used for each panel can be found in the Data Source file.

Supplementary Figure 3

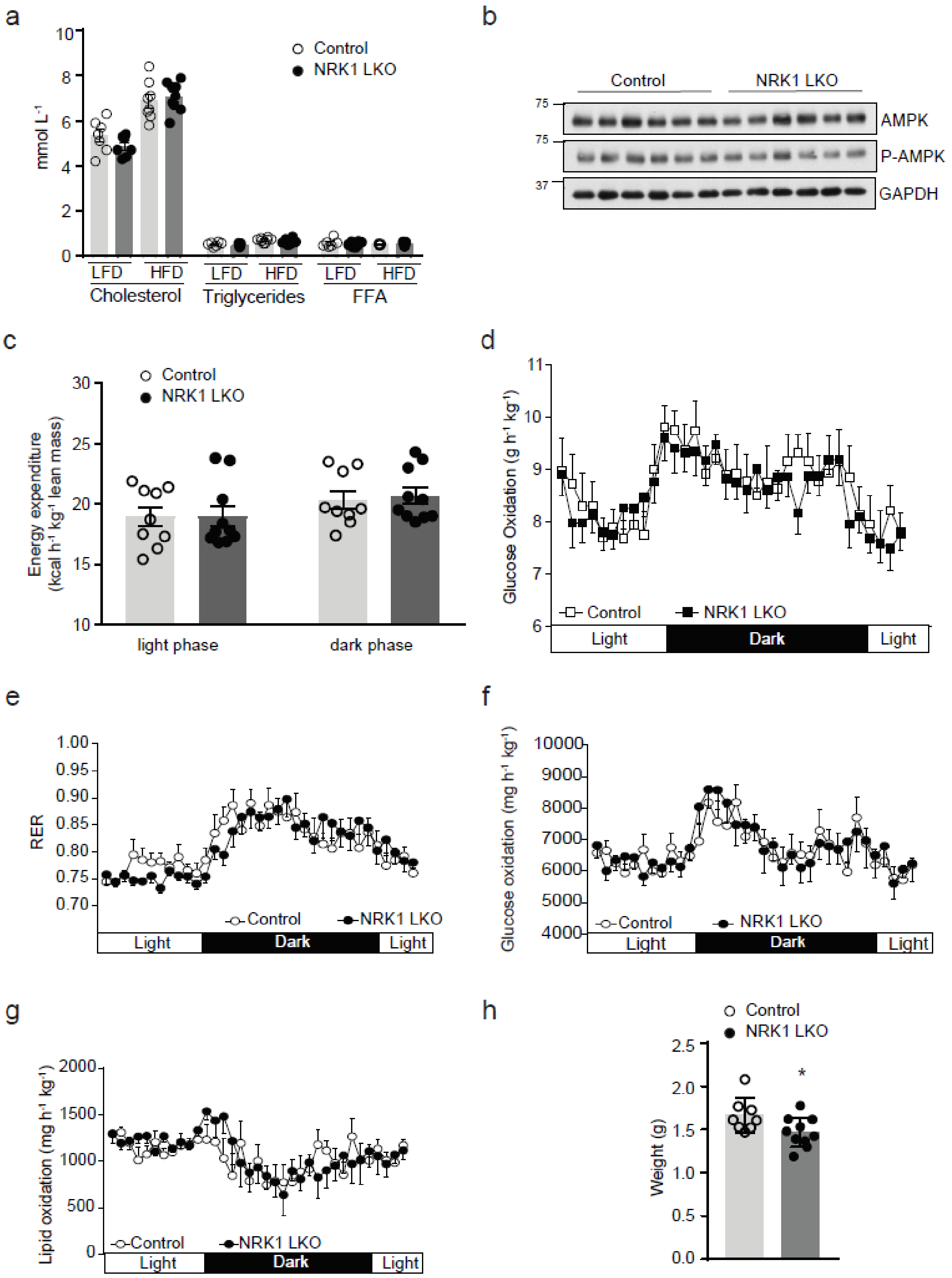

Supplementary Figure 3: Hepatic effects of NRK1 deficiency

a. Level of cholesterol, triglycerides and free fatty acids (FFA) in plasma from NRK1 LKO and control (Ctrl) mice on LFD (n=7 per group) and HFD (n=8 per group). **b**. Protein level of

AMPK, P-AMPK, and GAPDH in the liver of NRK1 LKO and Ctrl mice on HFD. **c**. Energy expenditure in NRK1 LKO (n=10) and control (n=9) mice on LFD. **d**. Glucose oxidation in NRK1 LKO (n=10) and control (n=9) mice on HFD. Data calculated from indirect calorimetry measurements: glucose oxidation = (4.55 x VCO2) – (3.21 x VO2). **e**. Respiratory exchange ratio (RER) in NRK1 LKO (n=8) and control (n=9) mice on LFD. **f**. Glucose oxidation in NRK1 LKO (n=8) and control (n=9) mice on LFD. Data calculated from indirect calorimetry measurements: glucose oxidation = (4.55 x VCO2) – (3.21 x VO2). **g.** Lipid oxidation in NRK1 LKO (n=8) and control (n=9) mice on LFD. Data calculated from indirect calorimetry measurements: lipid oxidation = (1.67 x VO2) – (1.67 x VCO2). **h.** Epididymal white adipose tissue (Epi WAT) weight of NRK1 LKO (n=10) and control (n=8) mice on HFD. Results shown are mean ± SEM, * $p<0.05$ ** $p<0.01$ *** $p<0.001$. The individual values and statistical tests used for each panel can be found in the Data Source file.

Supplementary Figure 4

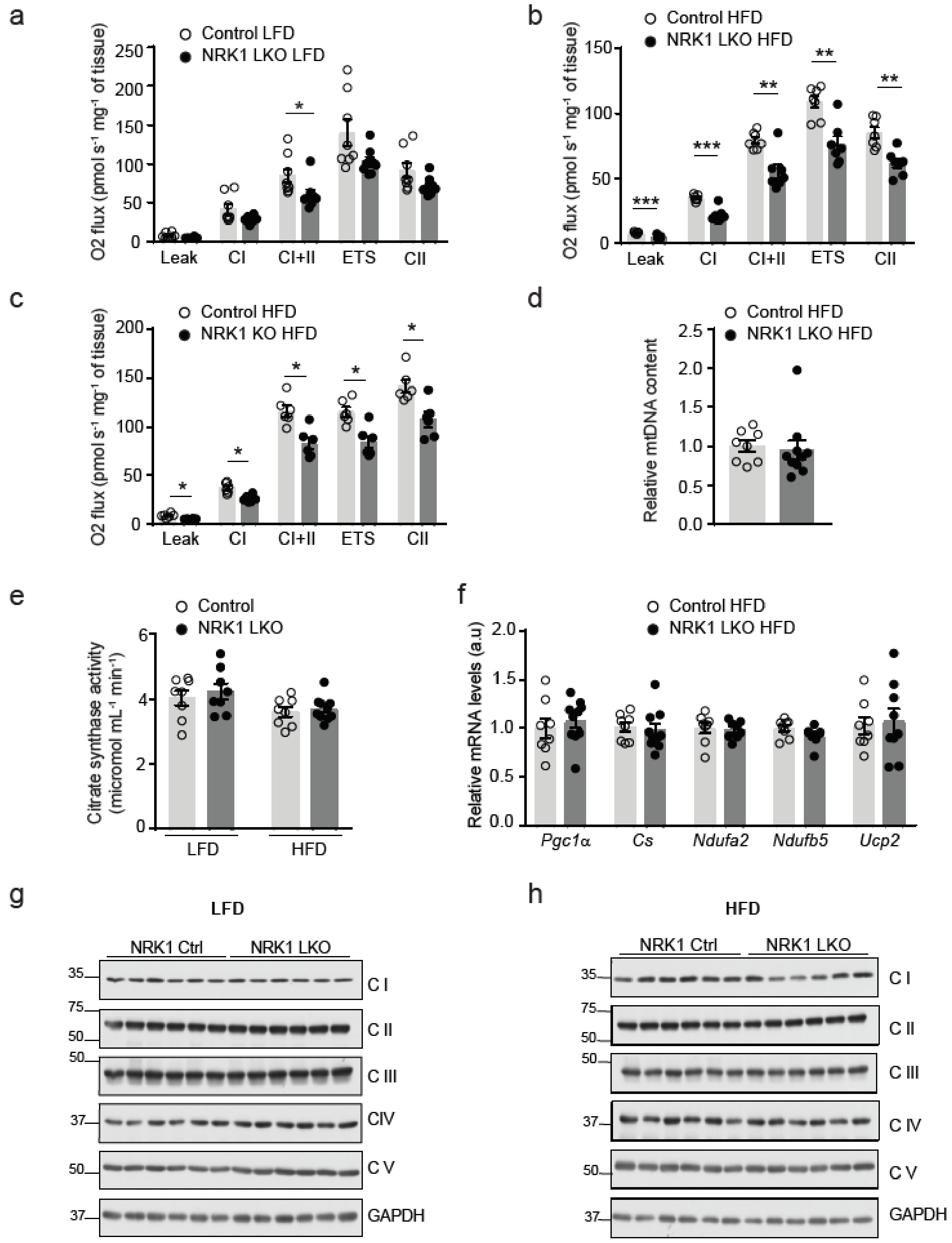

Supplementary Figure 4: Liver-specific NRK1 deficiency impairs mitochondrial respiration.

a-b. Mitochondrial respiration in liver homogenates of NRK1 LKO and control mice on LFD **(a)** and HFD **(b)** after a 6-hr fasting (n=8 and 7 mice, respectively, per group). **c**. Mitochondrial

respiration in liver homogenates of NRK1 total KO and control mice on HFD after a 6-hr fasting (n=6 mice per group). **d**. Relative mitochondrial DNA content in NRK1 LKO (n=9) and control (n=8) mice on HFD. **e**. Citrate synthase activity in liver homogenates from of NRK1 LKO and control mice on LFD and HFD (n=8 for all groups, except NRK1 LKO on HFD, n=10). **f**. Expression of mitochondrial genes in NRK1 LKO (n=10) and control (n=8) littermates on HFD. **g-h**. Mitochondrial protein level of Complex I (CI), Complex II (CII), Complex III (CIII), Complex IV (CIV), Complex V (CV) and GAPDH in the liver of NRK1 LKO and control mice on LFD **(g)** and HFD **(h)** (n=6 mice per group). Results shown are mean ± SEM; * $p<0.05$ ** $p<0.01$ *** $p<0.001$. The individual values and statistical tests used for each panel can be found in the Data Source file.

Supplementary Figure 5

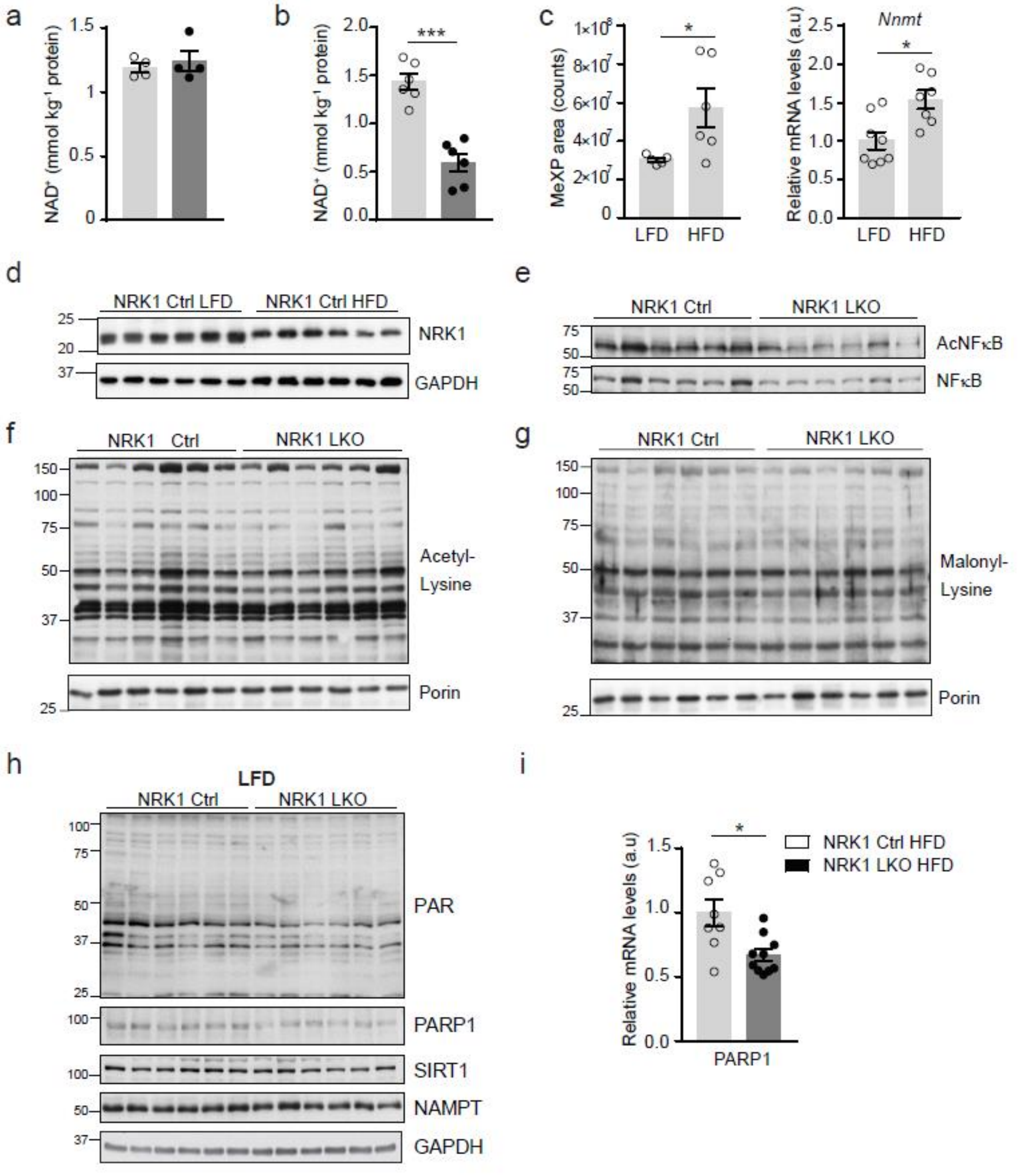

Supplementary Figure 5: NRK1 deletion represses PARP1 activity.

a. NAD$^+$ levels in the mitochondrial fraction of the liver of NRK1 LKO and control mice upon HFD (n=4 mice per group). **b**. NAD$^+$ levels in primary hepatocytes isolated from 8-12 week-old WT and NRK1 KO mice in condition of lipid overload (n=6 independent experiments). **c**.

MeXP metabolites (left) and *Nnmt* mRNA (right) levels in the liver of control mice upon LFD (n=5) and HFD (n=6). **d**. Protein level of NRK1 and GAPDH in the liver of control (WT) mice on LFD and HFD (n=6 mice per group). **e**. NF-κB acetylation level compared to total NF-κB in the liver of NRK1 LKO and control mice (n=6 mice per group). **f-g**. Total mitochondrial acetylation **(e)** and malonylation **(f)** level in the liver of NRK1 LKO and control mice on HFD. Porin was used as a loading control (n=6 mice per group). **h**. Levels of poly(ADP-ribose) protein modification, PARP1, SIRT1, NAMPT and GAPDH in the liver of NRK1 LKO and control mice fed with LFD. (n=6 mice per group). **i**. Gene expression of PARP1 in the liver of NRK1 LKO (n=9) and control (n=8) mice on HFD. Results shown are mean ± SEM, * $p<0.05$ ** $p<0.01$ *** $p<0.001$. The individual values and statistical tests used for each panel can be found in the Data Source file.

Supplementary Figure 6

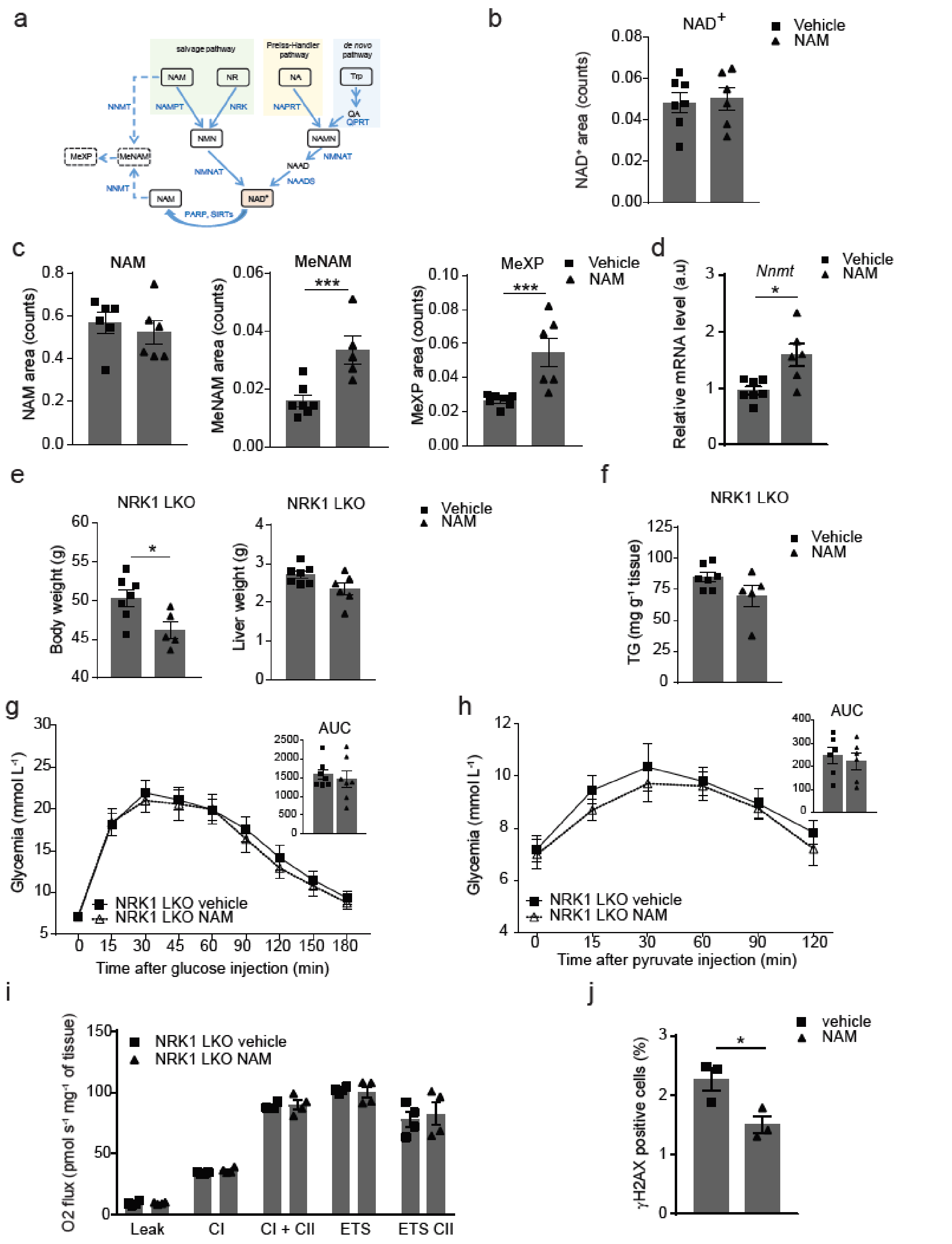

Supplementary Figure 6: NAM does not rescue the hepatic defects of NRK1 LKO mice.

a. Scheme representing NAD$^+$ biosynthesis pathways from the different NAD$^+$ precursors and NAD$^+$ consuming enzymes. **b**. NAD$^+$ levels in the liver of NRK1 LKO on HFD upon

supplementation with NAM (n=6) or vehicle (n=7). **c**. NAM (left), MeNAM (center) and MeXP (right) levels in the liver of NRK1 LKO on HFD upon supplementation with NAM (n=6) or vehicle (n=7). **d**. N*nmt* gene expression level in the liver of NRK1 LKO on HFD upon supplementation with NAM (n=6) or vehicle (n=7). **e**. Body weight (left) and liver weight (right) liver of NRK1 LKO on HFD upon supplementation with NAM (n=6) or vehicle (n=7). **f**. Triglycerides (TG) levels in liver of NRK1 LKO on HFD upon supplementation with NAM (n=5) or vehicle (n=7). **g-h**. Blood glucose level during intraperitoneal glucose (**g**) and pyruvate (**h**) tolerance test in NRK1 LKO on HFD upon supplementation with NAM (n=7 mice per group for ipGTT; n=6 mice per group for PTT). **i**. Mitochondrial respiration in the liver of NRK1 LKO on HFD upon supplementation with NAM (n=6) or vehicle (n=7). **j**. Quantification of γH2AX immunofluorescence on liver sections from NRK1 LKO on HFD upon supplementation with NAM or its vehicle (10x magnification, DAPI counterstaining, n=3 per group). Results shown are mean ± SEM, * $p<0.05$ ** $p<0.01$ *** $p<0.001$. The individual values and statistical tests used for each panel can be found in the Data Source file.

Supplementary Figure 7

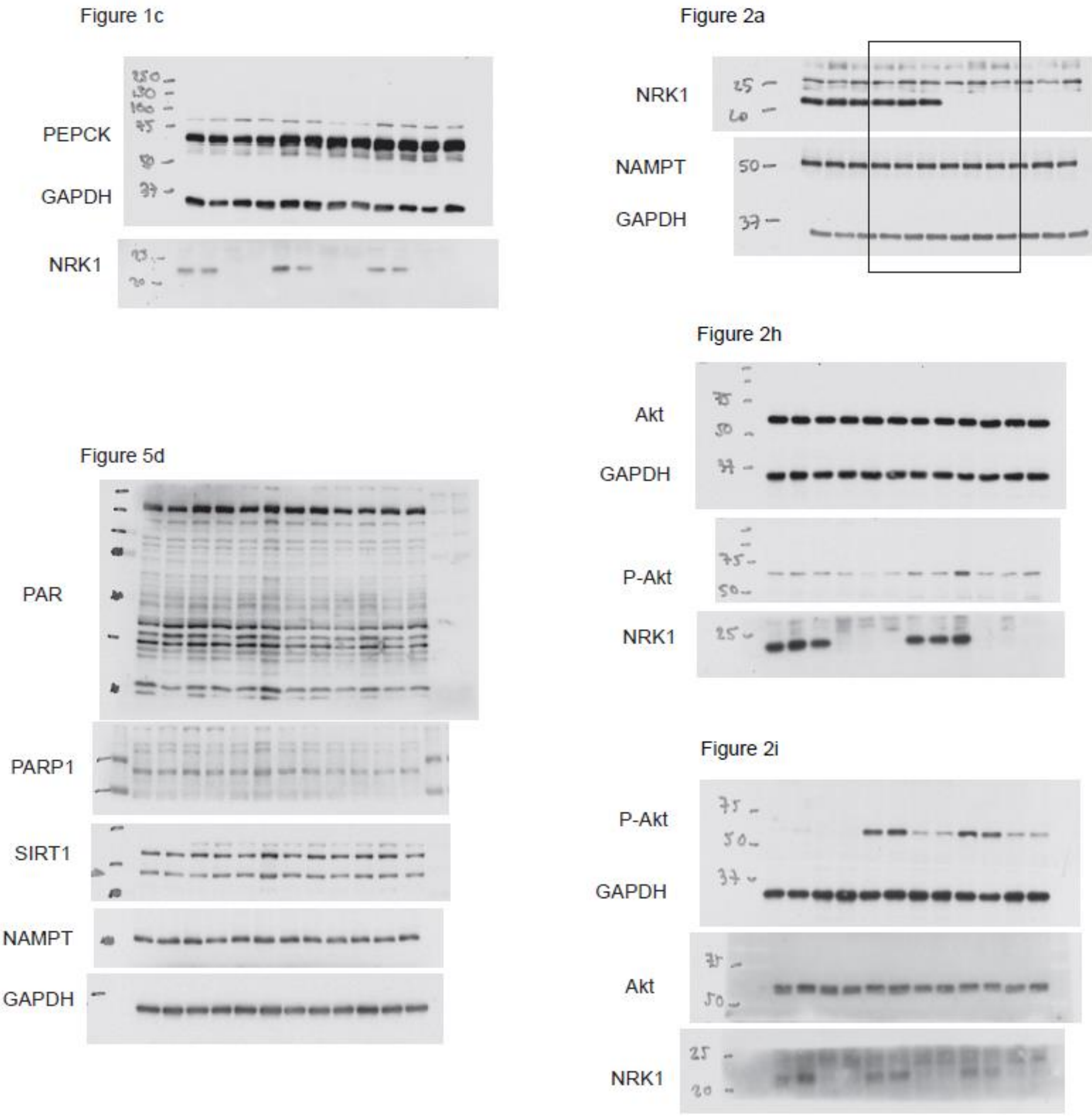

Supplementary Figure 7: Uncropped images from the main figures

Supplementary Figure 8

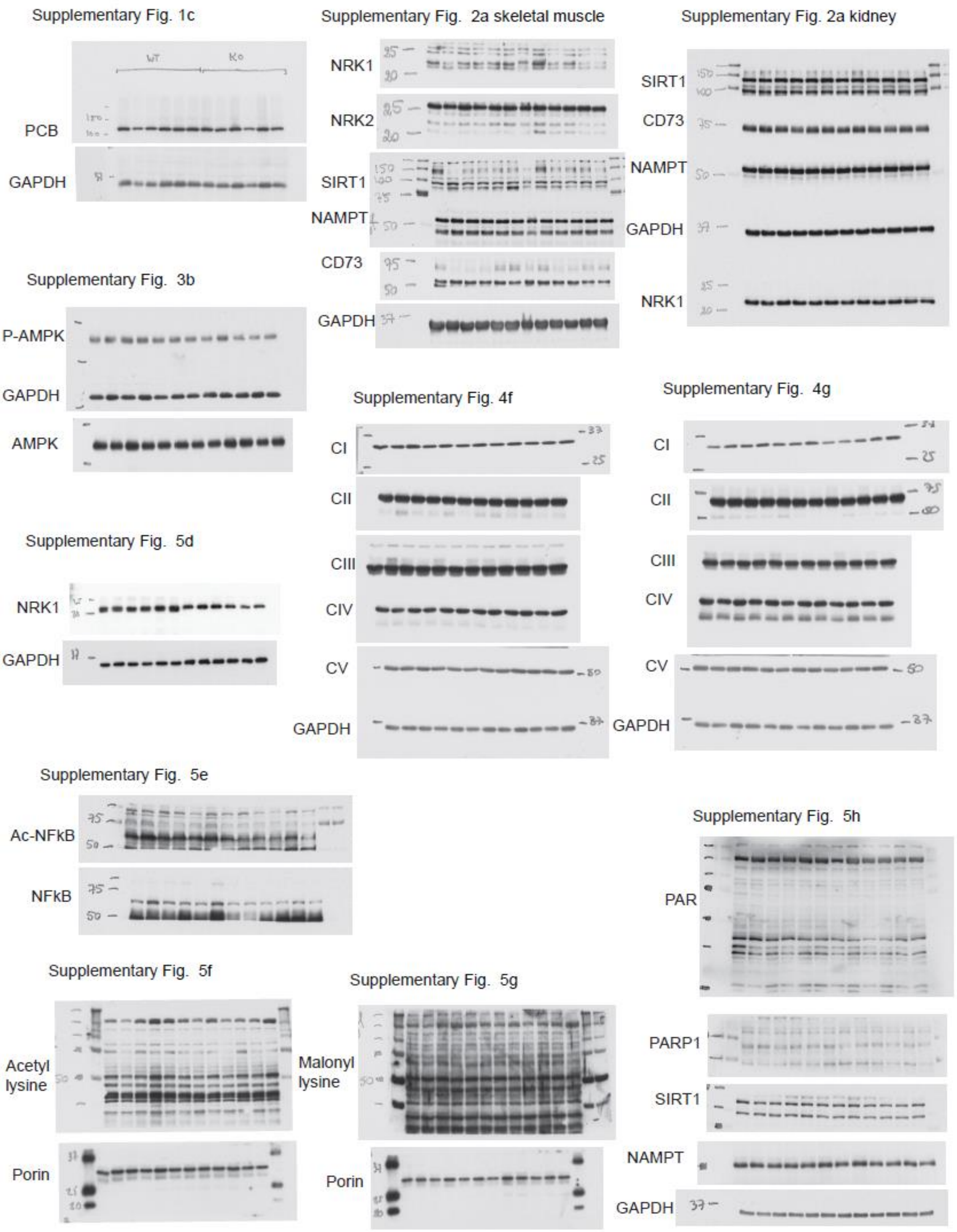

Supplementary Figure 8: Uncropped images from supplementary figures

Supplementary Figure 9

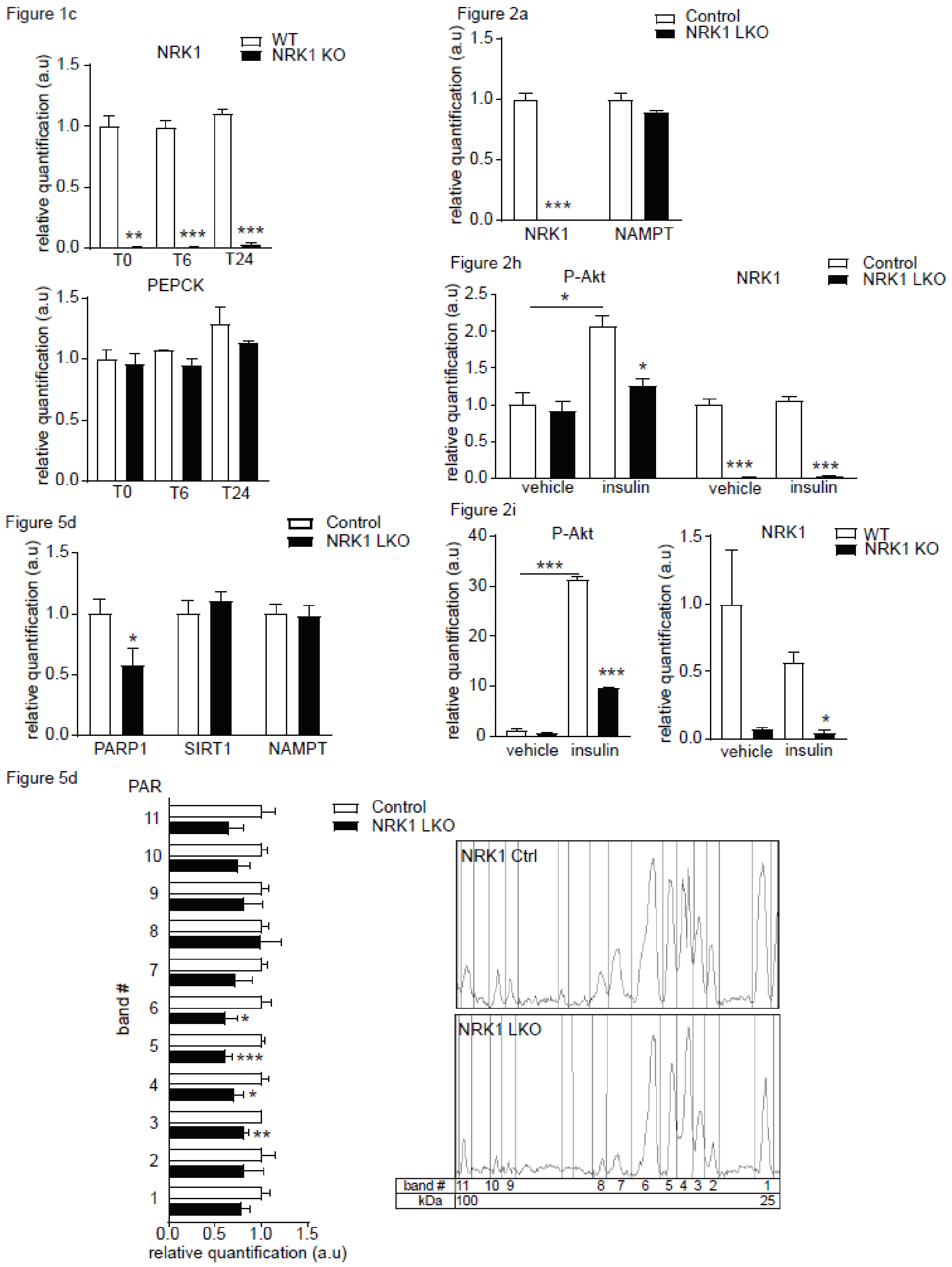

Supplementary Figure 9: Western blot quantifications for main figures

Supplementary Figure 10

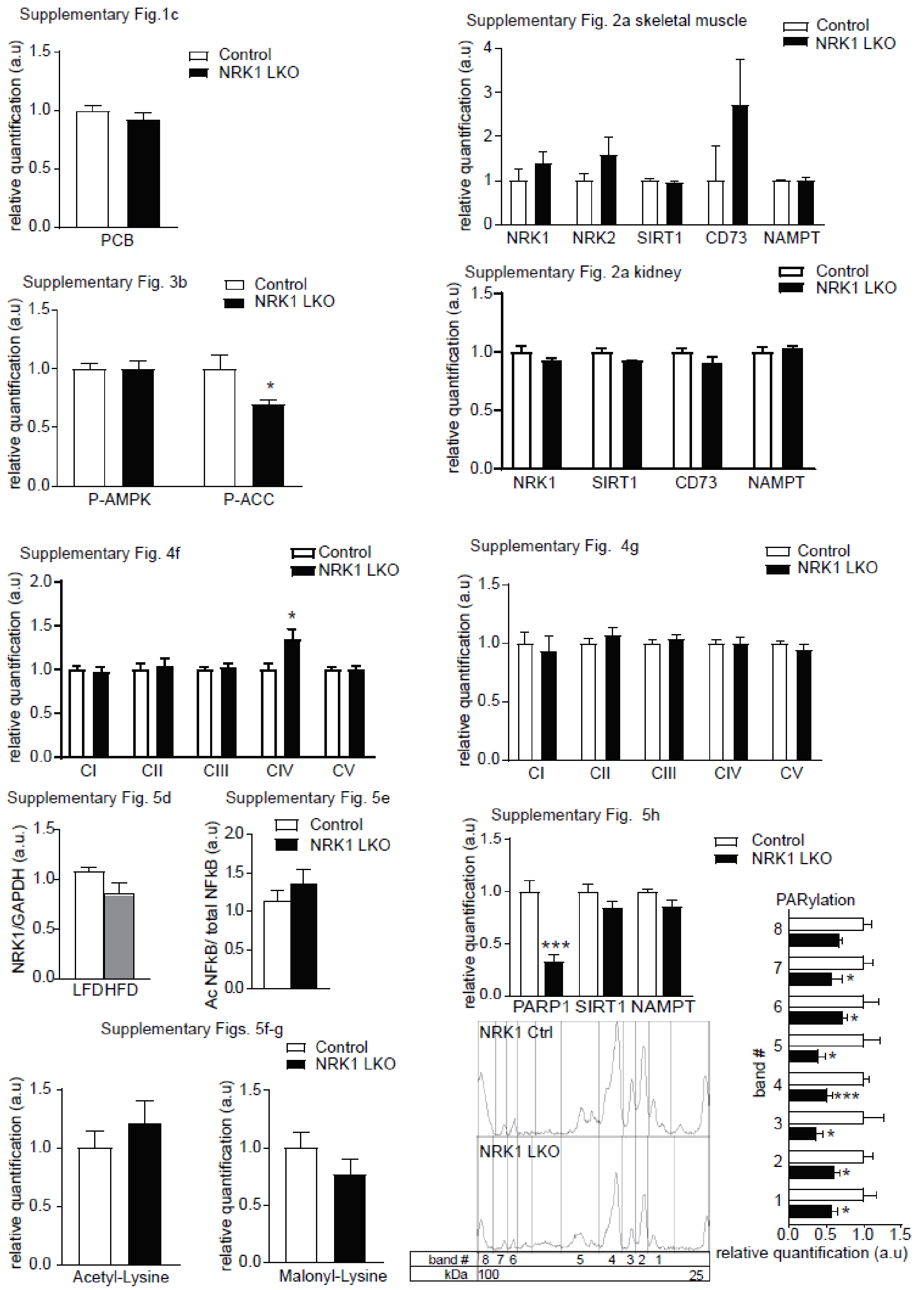

Supplementary Figure 10: Western blot quantifications for Supplementary Figures

Supplementary Table 1

Gene	Forward primer	Reverse primer
ApoB	TTGGCAAACTGCATAGCATCC	TCAAATTGGGACTCTCCTTTAGC
ApoE	ACAGATCAGCTCGAGTGGCAAA	ATCTTGCGCAGGTGTGTGGAGA
aSma	ACTGGGACGACATGGAAAAG	GTGCCTCTGTCAGCAGTGTC
B2m	ATG GGA AGC CGA ACA TAC TG	CAG TCT CAG TGG GGG TGA AT
Cd36	GATGTGGAACCCATAACTGGATTCAC	GGTCCCAGTCTCATTTAGCCACAGTA
CS	GGAGCCAAGAACTCATCCTG	TCTGGCCTGCTCCTTAGGTA
Colla1	TGTTCAGCTTTGTGGACCTC	GCAGCTGACTTCAGGGATGT
Cox2	GTTGATAACCGAGTCGTTCTGC	CCTGGGATGGCATCAGTTTT
Cpt1b	CCCATGTGCTCCTACCAGAT	CCTTGAAGAAGCGACCTTTG
Cpt2	AGCCAGTTCAGGAAGACAGA	GACAGAGTCTCGAGCAGTTA
Cxcl10	CCAAGTGCTGCCGTCATTTTC	GGCTCGCAGGGATTTCAA
Cyclophilin	CAGGGGAGATGGCACAGGAG	CGGCTGTCTGTCTTGGTGCTCTCC
Fas	TTCCAAGACGAAAATGATGC	AATTGTGGGATCAGGAGAGC
Fibronectin	ATGTGGACCCCTCCTGATAGT	GCCCAGTGATTTCAGCAAAGG
G6Pase	CCGGATCTACCTTGCTGCTCACTTT	TAGCAGGTAGAATCCAAGCGCGAAAC
Hk2	GCCAGCCTCTCCTGATTTTAGTGT	GGGAACACAAAAGACCTCTTCTGG
Hmgcr	CATCATCCTGACGATAACGCG	AGGCCAGCAATACCCAGAATG
Icam1	CGTGTGCCATGCCTTTAGCT	TCCAGTTATTTTGAGAGTGGTACAGTACTG
Ido2	CCTCATCCCTCCTTCCTTTC	GGAGCAATTGCCTGGTATGT
Il1b	GCAACTGTTCCTGAACTCAACT	ATCTTTTGGGGTCCGTCAACT
Lcad	GTAGCTTATGAATGTGTGCAACTC	GTCTTGCGATCAGCTCTTTCATTA
Ldlr	AGGCTGTGGGCTCCATAGG	TGCGGTCCAGGGTCATCT
Lrp	GGCTGCTGATGGCTCCCGAC	TCTCGTCCGTGCTGGCCAGG
Lxr	TGCCATCAGCATCTTCTCTG	GGCTCACCAGCTTCATTAGC
Mcad	GGCCATTAAGACCAAAGCAGA	GTGTCGGCTTCCACAATGAAT
Mcp1	GGAAGGCTTAATTGCAGCCA	TTCAGCCTTGCCATCTGCAT
Mttp1	GGAAGGCTTAATTGCAGCCA	TTCAGCCTTGCCATCTGCAT
Nampt	AGTGGCCACAAATTCCAGAGA	CCGCCACAGTATCTGTTCCTT
Naprt1	AGCCTCGTAGCCACCAATG	CTGCAATCAAGCGTAGCCG
Ndufa2	GCACACATTTCCCCACACTG	CCCAACCTGCCCATTCTGAT
Ndufab5	CTTCGAACTTCCTGCTCCTT	GGCCCTGAAAAGAACTAG
Nmnat1	TGGCTCTTTTAACCCCATCAC	TCTTCTTGTACGCATCACCGA
Nmnat3	TCACCCGTCAATGACAGCTAT	CACCCGAATCCAGTCAGATGT
Nnmt	AGCTTTGGGTCCAGACACTGT	GAGCCAATGTCAATCAGGAGTT
Pai-1	TCAGCCCTTGCTTGCCTCAT	GCATAGCCAGCACCGAGGA
Parp1	AAAAGGTGACGAGGTGGATG	ACCTGCTGCTGGTTGAAGAT
Pepck	CTG CAT AAC GGT CTG GAC TTC	CAG CAA CTG CCC GTA CTC C
Pgc1a	AAGTGTGGAACTCTCTGGAACTG	GGGTTATCTTGGTTGGCTTTATG
Ppara	AGGAAGCCGTTCTGTGACAT	TTGAAGGAGCTTTGGGAAGA
Pparg	ATGGGTGAAACTCTGGGAGATTCT	CTTGGAGCTTCAGGTCATATTTGTA
Qprt	CCGGGCCTCAATTTTGCATC	GGTGTTAAGAGCCACCCGTT
Scd1	ATGTCTGACCTGAAAGCCGA	GAAGGTGCTAACGAACAGGC
Srebp1c	ACCGTCACTTCCAGCTAGAC	CCACTAAGGTGCCTACAGAGC
Tdo1	AACATGCTCAAGGTGATAGCTC	GAACCGAGAACTGCTGTACCA
Tdo2	AGGAACATGCTCAAGGTGATAGC	CTGTAGACTCTGGAAGCCTGAT
Tnfa	GTAGCCCACGTCGTAGCAAAC	AGTTGGTTGTCTTTGAGATCCATG
Ucp2	CTACAGATGTGGTAAAGGTCCGC	GCAATGGTCTTGTAGGCTTCG
Vcam1	ACCTGTGCACAGCAACATGTG	GATCCTTGGGGAAAGAGTAGATGT C
16s	CCGCAAGGGAAAGATGAAAGAC	TCGTTTGGTTTCGGGGTTTC

Supplementary Table 1: List of primers used for qPCR (related to Methods)

Supplementary Table 2

Antibody	Reference
Acetyl-lysine	Cell signaling #9441
Ac-NFκB	Abcam ab52175
AMPK	Cell signaling #2532
Akt	Cell signaling #9272
CD45	Abcam ab10558
CD73	Cell signaling #13160
Complex I	Mitosciences ab14713
Complex II	Abcam ab14715
Complex III	Abcam ab190360
Complex IV	Abcam ab14705
Complex V	Abcam ab14748
GAPDH	Cell signaling #2118
Malonyl-lysine	PTM Biosciences PTM-901
NAMPT	Bethyl A300-372A
NFκB	Santa Cruz sc-8008
NRK1	(Ratajczak et al., 2016)
NRK2	(Ratajczak et al., 2016)
P-AMPK	Cell signaling #2531
P-Akt	Cell signaling #9271
PARP1	Santa Cruz sc-1561
PCB	Abcam ab126707
PEPCK	Abcam ab70358
PAR (Poly ADP-ribose)	Enzo ALX-210-890
Porine	Mitosciences ab14734
Sirt1	Cell signaling #2028

Supplementary Table 2: List of antibodies used for Western blot (related to Methods)

Supplementary Methods:

Animal phenotyping

Body weight was measured weekly. Body composition was determined by Echo-MRI (Echo Medical Systems). Oxygen consumption (VO_2), respiratory exchange ratio (RER), food intake and activity levels were monitored by indirect calorimetry using the comprehensive laboratory animal monitoring system (Columbus Instruments). Glucose and pyruvate tolerance tests were performed after overnight fasting by measuring glycemia after an intraperitoneal injection of 2 g/kg glucose or pyruvate (1g/kg for rescue experiment), respectively. For the glycerol tolerance test, 1 g/kg glycerol was injected intraperitoneally after overnight fasting. Insulin tolerance and insulin response were measured after a 6 hour-fasting period following injection of 0.5 U/kg or 1.0 U/kg in animals fed with LFD or HFD, respectively. Insulinemia was measured in plasma samples with an ELISA kit (EMD Millipore Corp.). Unless otherwise stated, all animals were euthanized after 2 hours of fasting using isoflurane. For fasting /refeeding experiment, food was removed for 24 hours. Then, the fasted group was immediately euthanized while the refeeding group was given access to food for another 6 hours. For the rescue experiment by NAM supplementation, mice were on HFD for 8 to 12 wks and drinking water was supplemented with NAM (Sigma #72340) (3g/L). Tissues were collected and flash-frozen in liquid nitrogen. Blood samples were collected in EDTA-Ca^{2+} tubes, and plasma was isolated after centrifugation. Plasma content of total cholesterol, triglycerides (TG), free fatty acid (FFA), alanine transaminase (ALT) and aspartate transaminase (AST) were measured using a Cobas c111 (Roche).

Western blotting

Protein extraction, quantification and western blotting procedures were performed as described previously (Ratajczak et al., 2016). All primary antibodies used are listed in Table S2. Primary antibodies were used in 1:5000 dilution for GAPDH and 1:1000 dilution for others. Antibody

detection reactions were developed by enhanced chemiluminescence (Amersham). For quantification, the intensity of each band was determined by densitometry using ImageJ software, normalized to GAPDH unless otherwise stated and relative to control condition.

RNA extraction and qPCR

Total mRNA from all studied tissues or cells was extracted using TRIzol (Life Technologies) according to the manufacturer's instructions. Reverse transcription was performed using SuperScript II (Life Technologies) according to the manufacturer's protocol. Quantification of mRNA expression was performed using SYBR Green real time PCR technology (Roche) using a Light Cycler (Roche). Gene expression was normalized with b2-microglobulin and cyclophillin as housekeeping genes. Relative gene expression between genotypes was assessed using the $\Delta\Delta$Ct method. The primers used are provided in the Table S1.

Mitochondrial Isolation

Mitochondria were isolated from mouse livers, which were immediately rinsed in ice-cold mitochondrial isolation buffer (IB) (250 mM sucrose, 10 mM Tris-MOPS and 0.1 mM EGTA/Tris; adjusted to pH=7.4). Pieces of tissue were placed in 5 ml of ice-cold fresh IB, transferred to a glass potter and homogenized at 4°C using a Teflon pestle rotating at 1600 rpm (4 strokes). The homogenate was centrifuged at 600g for 10 min at 4°C and the supernatant was then centrifuged at 7'000g for 10 min at 4°C. After this step, the supernatant containing cytosolic fraction was discarded and the pellet was washed once more with 5 ml of ice-cold IB and centrifuged for 10 min at 4°C. The pellet, containing mitochondria, was resuspended in 100 µl of buffer. Mitochondrial protein concentration was then determined using the BCA assay (Pierce).

Primary hepatocyte isolation

Hepatocytes were isolated from WT and NRK1 KO mice by continuous recirculating perfusion of the mouse liver *in situ* with collagenase digestion as described previously [1]. Cells were plated in M199 containing 100U/ml penicillin G, 100 mg/ml streptomycin, 0.1% (w/v) BSA, 10% (v/v) FBS, 10 nM insulin, 200 nM triiodothyronine and 100 nM dexamethasone. Post-attachment (4–5 h), cells were cultured overnight in M199 supplemented with antibiotics and 100 nM dexamethasone before used in experiments the following morning. For insulin stimulation, hepatocytes were treated for 15min with Insulin (30nM) or vehicle. To mimic lipid overload, culture medium was further supplemented with Oleic acid and palmitate (0.2mM each) and NR (1mM).

Fatty Acid Oxidation

Hot medium was prepared by adding 20 µl of 1 mM palmitate conjugated with 10% BSA and 0.5 µl of [9,10-^{3}H]-palmitic acid (NET043, PerkinElmer) to 1 ml of M199 low-glucose medium. Primary hepatocytes cultured in 12-well plates were washed with PBS before 1ml of hot medium was added per well. For negative control, 100 µM etomoxir was used. Plates were incubated for 3 hours at 37°C. Then, 200 µl of medium from samples was added to 800 µl of activated charcoal and the samples were incubated at room temperature with agitation for 30 min for absorption of unmetabolized palmitate. Blank sample was prepared from 200 µl of hot medium (without cells) processed similarly to the rest of the samples. After centrifugation for 15 min at 13’000 rpm, 0.2 ml of supernatant was mixed with 2.8 ml scintillation liquid and signal was measured for 5 min. In the assay, the amount of ^{3}H-labeled water formed from palmitate oxidation in the cell was measured. Signal was normalized for mg of protein and values from etomoxir-treated cells were subtracted from respective samples.

Analysis of NR, NAM, MeXP and NMN by LC-MS

Sample preparation for metabolomics analysis was based on[2]. In short, frozen samples were extracted in 1300 µL of a cold mixture of methanol:water:chloroform in 5:3:5 (v/v) proportion with 5 µM of internal standard NAM-d4, while keeping the samples cold throughout the procedure. Liver extracts were homogenized with 3 mm tungsten carbide beads using a tissue mixer (Qiagen TissueLyser II) for 1.5 min at 20 Hz, followed by 10 min 1500 rpm shaking at 4°C in a thermo-shaker (Thermomixer C, Eppendorf). Samples were then centrifuged 10 minutes 15,000 rpm at 4°C, resulting in two phases: upper phase containing the polar metabolites and lower phase containing apolar metabolites, whilst in the middle a protein layer remained. The protein layer was taken for quantification using the bicinchoninic acid (BCA) assay (ThermoFisher Scientific). The upper phase was dried overnight in a vacuum centrifuge at 4°C and 5 mbar, and then stored at -80°C, before metabolomics analysis. Dry samples were reconstituted in 50 µL 60% (v/v) acetonitrile:water, centrifuged for 2 minutes at 15,000 rpm, and the supernatant transferred into a glass vial for hydrophilic interaction ultra high performance liquid chromatography mass spectrometry (UHPLC-MS) analysis. The UHPLC consisted of a binary pump, a cooled autosampler, and a column oven (Vanquish UHPLC+ Focused, Thermo Scientific), connected to an orbitrap mass spectrometer (Orbitrap Fusion Lumos Tribrid, Thermo Scientific) equipped with a heated electrospray ionisation (H-ESI) source. Of each sample 2 µL were injected into the analytical column (2.1 mm x 150 mm, 5 µm pore size, 200 Å HILICON iHILIC®-Fusion(P)), guarded by a pre-column (2.1 mm x 20 mm, 200 Å HILICON iHILIC®-Fusion(P) Guard Kit) operating at 35 °C. The mobile phase (10 mM ammonium acetate at pH 9, A, and acetonitrile, B) was pumped at 0.25 mL/min flow rate over a linear gradient of decreasing organic solvent (0.5-16 min, 90-25% B), followed by re-equilibration for a total running time of 30 min. On-the-fly alternating negative (3 kV) and positive (3.5 kV) ion modes were used and the following source parameters were applied: 20

sheath gas, 15 aux gas, ion transfer tube temperature 310 °C, and vaporizer temperature 280 °C. MS acquisition was performed at 60k orbitrap resolution, in centroid mode, by scanning between the mass range 85 to 850 Da. The software Xcalibur v4.1.31.9 (Thermo Scientific) was used for instrument control, data acquisition and processing. Positive ion mode extracted chromatograms using the corresponding accurate mass of NR, nicotinamide (NAM), NMN and MeXP (combination of either/or N-methyl-2-pyridone-5-carboxamide (Me2PY) and N-methyl-4-pyridone-carboxamide (Me4PY)) were integrated and used for relative comparison, normalised to internal standard and total protein. Me2PY and Me4PY, being conformational isomers, have the same mass and could not be separated by our chromatography, thus the naming MeXP. Retention time and mass detection of metabolites was confirmed by authentic standards.

Triglycerides content

Triglycerides were measured in liver tissue using the Bioassay System Assay Kit (ETGA-200) according to manufacturer's instructions.

NAD^+ assay

NAD^+ was extracted from tissues and measured by EnzyChrom NAD/NADH Assay Kit (BioAssay Systems) according to manufacturer's instructions.

Citrate synthase activity assay

Citrate synthase activity was assessed in liver tissue homogenates using Citrate Synthase Assay kit (Sigma, CS0720) following manufacturer's instructions.

Mitochondrial DNA content

Genomic DNA was extracted from liver tissue using DNeasy Blood and Tissue kit (Qiagen) according to the manufacturer's instructions. Mitochondrial and nuclear DNA content were

determined by measuring 16s, COX2 and HK2, UCP2 respectively by quantitative PCR using SYBR Green and primers listed in table S1.

Supplementary references

1 Ratajczak, J. *et al.* NRK1 controls nicotinamide mononucleotide and nicotinamide riboside metabolism in mammalian cells. *Nature communications* **7**, 13103, doi:10.1038/ncomms13103 (2016).

2 Fendt, S. M. *et al.* Reductive glutamine metabolism is a function of the alpha-ketoglutarate to citrate ratio in cells. *Nature communications* **4**, 2236, doi:10.1038/ncomms3236 (2013).
